# Preliminary evidence of links between ayahuasca use and the corpus callosum

**DOI:** 10.3389/fpsyt.2022.1002455

**Published:** 2022-10-26

**Authors:** Otto Simonsson, José Carlos Bouso, Florian Kurth, Dráulio B. Araújo, Christian Gaser, Jordi Riba, Eileen Luders

**Affiliations:** ^1^Center for Psychiatry Research, Department of Clinical Neuroscience, Karolinska Institute, Stockholm, Sweden; ^2^Department of Sociology, University of Oxford, Oxford, United Kingdom; ^3^ICEERS–International Center for Ethnobotanical Education, Research and Services, Barcelona, Spain; ^4^Medical Anthropology Research Center (MARC), Universitat Rovira i Virgili, Tarragona, Spain; ^5^Department of Neurosciences and Behavior, Ribeirão Preto Medical School, University of São Paulo, Ribeirão Preto, Brazil; ^6^School of Psychology, University of Auckland, Auckland, New Zealand; ^7^Brain Institute, Federal University of Rio Grande do Norte, Natal, Brazil; ^8^Onofre Lopes University Hospital, Federal University of Rio Grande do Norte, Natal, Brazil; ^9^Department of Psychiatry and Psychotherapy, Jena University Hospital, Jena, Germany; ^10^Department of Neurology, Jena University Hospital, Jena, Germany; ^11^Centro de Investigación Biomédica en Red de Salud Mental, Madrid, Spain; ^12^Department of Women’s and Children’s Health, Uppsala University, Uppsala, Sweden; ^13^Laboratory of Neuro Imaging, School of Medicine, University of Southern California, Los Angeles, CA, United States

**Keywords:** psychedelics, ayahuasca, neuro, brain, corpus callosum, isthmus

## Abstract

**Background:**

Recent research suggests that ayahuasca and its alkaloid-containing ingredients may be helpful in the treatment and prevention of certain movement and neurodegenerative disorders. However, such research is still in its infancy and more studies in normative samples seem necessary to explore effects of ayahuasca on clinically relevant brain structures, such as the corpus callosum.

**Aims:**

The purpose of the present study was to investigate links between ayahuasca use and callosal structure in a normative sample.

**Methods:**

Using structural imaging data from 22 ayahuasca users and 22 matched controls we compared the thickness of the corpus callosum between both groups at 100 equidistant points across the entire midsagittal surface. In addition, we investigated point-wise correlations between callosal thickness and the number of past ayahuasca sessions.

**Results:**

The corpus callosum was significantly thicker within the isthmus in the ayahuasca group than in the control group. There was also a significant positive correlation between callosal thickness and the number of past ayahuasca sessions within the rostral body, albeit none of these effects survived corrections for multiple comparisons. No region was significantly thicker in the control than in the ayahuasca group, and no callosal region was negatively linked to ayahuasca use, even at uncorrected significance thresholds.

**Conclusion:**

This study provides preliminary evidence of links between ayahuasca use and the corpus callosum. However, future studies need to replicate these findings, preferably using larger sample sizes and ideally also utilizing longitudinal research designs, to draw any practical conclusion and offer implications for follow-up clinical research.

## Introduction

Ayahuasca is a psychedelic botanical admixture that has been used for millennia by indigenous tribes in the Amazon basin. The most commonly known recipe for ayahuasca involves preparing a concoction of *Banisteriopsis caapi* and *Psychotria viridis*. The stem and bark of *B. caapi* contain the β-Carboline alkaloids harmaline, harmine, and tetrahydroharmine, while the leaves of *P. viridis* contain the psychoactive alkaloid N, N-Dimethyltryptamine (DMT), also known as DMT (1). If ingested orally, DMT becomes degraded by monoamine oxidase type A (MAO-A) in the gastrointestinal tract and therefore cannot reach the brain. However, when mixing *B. caapi* with *P. viridis* into an ayahuasca brew, reversible MAO-A inhibitors present in the β-Carboline alkaloids allows DMT to be delivered to the Central Nervous System (CNS), which produces potent psychoactive effects (2).

The evidence to date suggests that ayahuasca has a relatively good safety profile [(3); but also see (4, 5) for potential harmful effects]. While ayahuasca and its alkaloid-containing ingredients show promise in the treatment of mental health conditions (6–9), they might also aid in the treatment and prevention of movement and neurodegenerative disorders. For example, DMT binds and activates the sigma-1 receptor (10) as well as several serotonin receptors (11) which have been identified as potential therapeutic targets in certain movement and neurodegenerative disorders (12–15). Other research suggests that DMT and the alkaloids of *B. caapi* (harmine, tetrahydroharmine, harmaline) may stimulate adult neurogenesis (16–18) and reduce neuroinflammation (19). Ayahuasca might therefore become relevant for a variety of disorders, such as multiple sclerosis, amyotrophic lateral sclerosis, or Parkinson’s disease (19). While the etiology and disease progression varies across these types of disorders, many have been associated with aberrations of the corpus callosum (20–27), which is the largest interhemispheric fiber tract in the human brain (28).

Thus, the current study was designed to shed further light on the potential effects of ayahuasca use on the corpus callosum. More specifically, we conducted a secondary analysis [see (29) for the original study] comparing the midsagittal thickness of the corpus callosum between ayahuasca users and their matched controls at 100 equidistant points between the tip of the rostrum and the bottom of the splenium. In addition, we conducted point-wise correlation analyses to establish whether callosal thickness and the number of past ayahuasca sessions were significantly linked. We hypothesized thicker corpora callosa in ayahuasca users compared to matched controls as well as a positive correlation between callosal thickness and ayahuasca use. Since some research suggests that specific components of the ayahuasca brew may improve motor function [(30, 31); see also, (32)], we expected to find significant effects within the isthmus of the corpus callosum, which has been demonstrated to house motor fibers (33) and has been linked to certain movement and neurodegenerative disorders in previous research [(21, 34, 35); see also (36)].

## Materials and methods

### Sample

The study included 22 ayahuasca users from the Santo Daime church in Spain and 22 matched controls. The inclusion criteria for the ayahuasca group were: (1) frequent use of ayahuasca in the past 2 years; (2) no ayahuasca use or use of other types of drugs in the 2 weeks before the brain scan (verified by an urine toxicology test); (3) lifetime use of cannabis less than twenty times; (4) lifetime use of other drugs on ten occasions or less; and (5) no current or past history of psychiatric or neurological disorders [see (29) for a detailed overview of the full inclusion criteria]. The ayahuasca group and the control group were matched for sex (6 men and 16 women), age (ayahuasca group: mean = 40.9 years, SD = 12.6; control group: mean = 41.5 years, SD = 11.8), years of education (ayahuasca group: mean = 13.0 years, SD = 3.3; control group: 13.1 years, SD = 3.1), fluid intelligence as measured by the Wechsler Adult Intelligence Scale-III [(37); ayahuasca group: mean = 15.7, SD = 3.5; control group: 15.7, SD = 3.6], and verbal intelligence as measured by the Spanish version of the New Adult Reading Test [(38); ayahuasca group: mean = 25.9, SD = 3.5; control group: 25.0, SD = 3.7]. There were also no significant differences between the two groups with respect to employment status, marital status, tobacco use, and alcohol use. A more detailed explanation of the testing batteries and recruitment process can be found in Bouso et al. (29).

### Image acquisition and processing

The structural brain images were acquired on a 3 Tesla scanner using a T1-weighted MPRAGE sequence (240 sagittal slices; matrix size = 256 × 256; voxel resolution = 1 mm × 1 mm × 1 mm; TR = 2300 ms; TE = 1 ms). The study was approval by the Ethics Committee at Hospital de Sant Pau (Barcelona, Spain). All participants provided written informed consent to participate in the study.

All brain images were preprocessed in SPM12^1^ and the CAT12 toolbox (39) applying corrections for magnetic field inhomogeneities and spatial alignment, the latter using rigid-body transformations. Using the preprocessed images, the corpus callosum was manually outlined in each brain’s midsagittal section (40). The callosal traces were extracted and automatically processed in a number of successive steps (41–43). More specifically, the callosal outlines were separated into 100 nodes and re-sampled at regular intervals rendering the discrete points comprising the two boundaries spatially uniform. Then, a new midline curve was created by calculating the two-dimensional average from the 100 equidistant nodes representing the upper and the lower callosal boundaries. Finally, the distances between the 100 nodes of the upper as well as the lower callosal boundaries to the 100 nodes of the midline curve were calculated and later entered as the dependent variables in the statistical model. In addition, the total intracranial volume (TIV) was calculated by classifying images as gray matter (GM), white matter (WM), and cerebrospinal fluid (CSF) in native space and adding the volumes of these compartments (TIV = GM + WM + CSF) to be entered as a nuisance variable in the statistical model.

### Statistical analyses

All statistical analyses were conducted in Matlab^2^ using a mass-univariate general linear model. The calculated point-wise callosal distances constituted the dependent variable, group the independent variable, and age as well as TIV the variables of no interest. In addition to the group comparison (ayahuasca group vs. control group), we conducted a correlation analysis within the ayahuasca group examining the link between callosal thickness and the number of past ayahuasca sessions. Again, age and TIV were considered variables of no interest. For both analysis streams–group comparison and correlation–given our *a priori* hypotheses, we applied one-tailed *T*-tests at *p* ≤ 0.05 and generated uncorrected significance maps (effect size maps, respectively) by projecting the *p*-values (d- and r-vales, respectively) onto the averaged callosal outline. In addition, corrections for multiple comparisons were applied using a Monte-Carlo simulation with 10,000 permutations (44, 45) to test if findings survived a correction for multiple comparisons.

## Results

**FIGURE 1:**
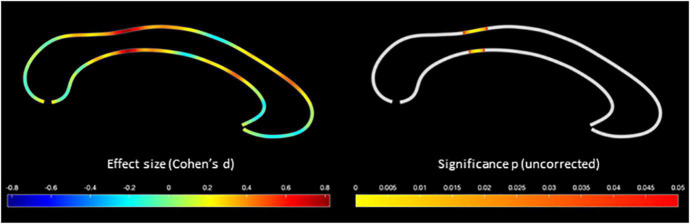
Greater callosal thickness in the ayahuasca group compared to the control group. The color bar encodes the effect size (Cohen’s d) on the **left**, and the uncorrected significance (p) on the **right**.

**FIGURE 2:**
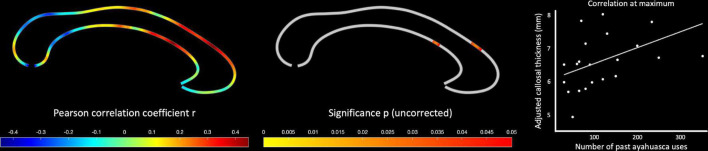
Positive correlations between callosal thickness and ayahuasca use. The color bar encodes the Pearson correlation coefficient (r) on the **left**, and the uncorrected significance (p) in the **middle**. The scatterplot on the **right** illustrates the association between the number of ayahuasca uses and callosal thickness at the maximum of the significance cluster, adjusted for age and total intracranial volume (TIV).

As shown in Figure 1, we detected significant group differences within the isthmus, where the corpus callosum was thicker in the ayahuasca group than in the control group. With respect to the significance maximum within this isthmus cluster, the mean (±SD) callosal thickness in the ayahuasca group was 5.47 (±0.71) mm and 4.78 (±1.01) mm in the control group (*p* = 0.006; *d* = 0.85). The ayahuasca group reported 123 past ayahuasca sessions on average (range: 30–352), and we observed a significant positive correlation between callosal thickness and the number of sessions within the rostral body (see Figure 2). With respect to the significance maximum within this rostral body cluster, the observed *p*-value was 0.026 and the correlation coefficient (r) was 0.45, which constitutes a medium effect size. None of these significant effects survived corrections for multiple comparisons. No region was significantly thicker in the control than in the ayahuasca group, and no callosal region was negatively linked to ayahuasca use, even without applying corrections for multiple comparisons.

## Discussion

This study investigated associations between long-term use of ayahuasca and the anatomy of the corpus callosum. The results showed that the isthmus of the corpus callosum was thicker in the ayahuasca group than in the control group, but there was also a positive correlation between callosal thickness and the number of past ayahuasca sessions within the rostral body. None of these findings survived a correction for multiple comparisons, however, and should therefore be interpreted with caution. Prior investigations comparing ayahuasca users with non-users have found group differences in neuropsychological functioning and neuroanatomy, albeit none in the corpus callosum (29, 46, 47). The preliminary and exploratory findings of this study therefore contribute to a growing body of research on the potential effects of ayahuasca on brain function and structure.

In terms of the location of the observed effects and their possible functional implications, both the isthmus and the rostral body of the corpus callosum have been linked to motor function (33, 48–51). With respect to the possible underlying mechanisms, previous research suggests that a thicker corpus callosum (i.e., as observed in the ayahuasca group) implies a higher number of axons, thicker axons, a greater degree of myelination, or possibly a combination of these (52). More axons might indicate greater anatomical interhemispheric connectivity, and thicker axons as well as a greater myelination are likely associated with higher signal conduction velocities [see (48, 53, 54)]. It is therefore possible that ayahuasca could have a modulating impact and possibly benefiting effect on symptoms linked to impaired motor function, such as those evident in many movement and neurodegenerative disorders (55–57).

### Limitations and implications for future research

The current pilot study has several short-comings. First, given that the structural brain images were only acquired at one time point, causality of the reported effects cannot be resolved. It is possible, for instance, that brains of long-term ayahuasca users are inherently different from non-users. Second, participants were asked about the number of past ayahuasca sessions, but no information was provided on the (average) dose of ayahuasca on each occasion. The analyses that were conducted could therefore not account for potential dose-dependent effects. Third, none of the significant effects survived corrections for multiple comparisons. Caution should therefore be exercised when interpreting the results. Last but not least, the study was conducted in healthy participants and not in patients affected by movement and neurodegenerative disorders. All conclusions with respect to potential treatments should therefore be drawn with extreme caution and considered conjecture.

Future research is clearly necessary to replicate the current findings as well as to address the aforementioned limitations by using longitudinal research designs, by collecting information on motor and potentially other behavioral or cognitive information, and by additionally investigating dose-dependent effects. Once a solid basis has been established, normative research can be taken into patient populations.

## Conclusion

The research on ayahuasca has primarily focused on mental health, but relatively little is known about the potential of ayahuasca and its alkaloid-containing ingredients in the treatment and prevention of movement and neurodegenerative disorders. Given that the corpus callosum has been implicated as a key structure in certain movement and neurodegenerative disorders, this study investigated links between ayahuasca use and the corpus callosum. The findings provide preliminary evidence of links between ayahuasca use and callosal structure, albeit a causality of the reported effects cannot be resolved given the cross-sectional design. Future studies, ideally longitudinal in nature, need to replicate the current findings in larger normative samples, which could be followed by clinical studies using actual patient populations.

## Data availability statement

The raw data supporting the conclusions of this article will be made available by the authors, without undue reservation.

## Ethics statement

The studies involving human participants were reviewed and approved by the Ethics Committee at Hospital de Sant Pau (Barcelona, Spain). The participants provided their written informed consent to participate in this study.

## Author contributions

JB, JR, and DA designed the study and were responsible for data acquisition. FK, CG, and EL analyzed the data. OS wrote the manuscript, with input from EL, FK, JB, DA, and CG. All authors contributed to the article and approved the submitted version.

## Footnotes

1
http://www.fil.ion.ucl.ac.uk/spm

2
https://au.mathworks.com/products/matlab.html
